# 1,25-D3 Protects Diabetic Brain Injury Through GLP-1R/PI3K/Akt Pathway by Experimental and Molecular Docking Studies

**DOI:** 10.1155/mi/8217035

**Published:** 2025-03-07

**Authors:** Ting Song, Bin Wang, Yutian Li, Yingzhe Zhao, Jian Li, Yanqiang Wang, Xiangling Li

**Affiliations:** ^1^Department of Neurology Ⅱ, The Affiliated Hospital of Shandong Second Medical University, Weifang, Shandong, China; ^2^School of Pharmacy, Shandong Second Medical University, Weifang, Shandong, China; ^3^Department of Internal Medicine, The Affiliated Hospital of Shandong Second Medical University, Shandong Second Medical University, Weifang, Shandong, China

**Keywords:** 1,25-D3, diabetic brain injury, GLP1-R/PI3K/AKT, neuroinflammation, vascular dysfunction

## Abstract

**Background:** Diabetes can cause an increase in intracellular glucose, leading to neuronal damage and microvascular dysfunction. Neuroprotective agents 1*α*,25-dihydroxyvitamin D3 (1,25-D3) can reduce neurological complications. The main purpose of this study is to evaluate the levels of inflammatory factors and vascular protective factors in streptozotocin (STZ)-induced diabetic rats and determine whether 1,25-D3 can protect the rat brains from hyperglycemia through the glucagon-like peptide-1 (GLP-1)R/PI3K/AKT signal pathway.

**Methods:** We first evaluated whether the relevant target could effectively bind to 1,25-D3 through molecular docking. Next, we established STZ-induced diabetic rat models for in vivo experiments to verify the targets in molecular docking that have good binding effects on 1,25-D3. After 8 weeks of a high-fat diet (HFD) and an intraperitoneal injection of STZ (35 mg/kg body weight), the experimental type 2 diabetic rat model was created, and the morphological changes of the cerebral cortex were measured by performing hematoxylin and eosin (H&E) staining. Western blotting (WB) was used to detect the proteins' expression of relevant targets, and the RT-qPCR was used to analyze the mRNA levels of relevant targets in the cerebral cortex. We also utilized the enzyme-linked immunosorbent assay (ELISA) kit for detecting the protein content of relevant targets.

**Results:** Molecular docking showed that 1,25-D3 had good binding ability with related targets, such as GLP-1R, PI3K, AKT1, vascular endothelial growth factor-*α* (VEGF-*α*), endothelial nitric oxide (NO) synthase (e-NOS), intercellular adhesion molecule-1 (ICAM-1), and vascular intercellular adhesion molecule-1 (VCAM-1). Experimental verification results found that 1,25-D3 partially prevented abnormalities in brain function and structure caused by diabetes. Meanwhile, the ICAM-1 and VCAM-1 levels were increased in the high-glucose group, e-NOS levels were decreased, and the relative expression of GLP-1R, VEGF-*α*, p-PI3K/PI3K, and p-AKT/AKT was reduced. 1,25-D3 abolished these changes, and these effects were suppressed by specific inhibitors.

**Conclusions:** 1,25-D3 alleviates neuroinflammation and improves vascular endothelial dysfunction through multitarget and multipathway by upregulating the GLP-1R/PI3K/AKT signaling axis to improve diabetes-induced brain injury.

## 1. Introduction

Diabetes can lead to various cardiovascular and cerebrovascular complications, posing significant risks to patient survival [1]. The pathophysiological mechanisms underlying hyperglycemia-induced brain damage are multifaceted, involving oxidative stress, vascular dysfunction, neuroinflammation, neurodegeneration, and mitochondrial impairment, among other processes [2]. Ongoing research continues to explore neuroprotective and restorative therapies targeting these mechanisms in diabetic brain injury. Notably, recent studies have identified the potential of several noninsulin pharmacologic agents, including antioxidants and neuroinflammation inhibitors, which may offer therapeutic benefits for mitigating diabetes-related brain damage.

1*α*,25-Dihydroxyvitamin D (3) (1,25-D3) [1*α*,25(OH)2-D3], (1,25(OH) (2)D (3)), (1,25-D3), a promising neuroprotective agent, supports vascular function by inhibiting neuroinflammation, modulating endocrine activity, influencing angiogenesis, and activating various biological pathways [3–5]. Experimental studies suggest that 1,25-D3 interacts with the vitamin D receptor (VDR) to exert antioxidant and anti-inflammatory effects, thereby preserving mitochondrial and neuronal cell functions [6]. In recent years, these attributes have led to the growing application of 1,25-D3 in the treatment of neurological disorders, with no serious side effects reported. This underscores the substantial therapeutic potential of 1,25-D3. Emerging evidence also indicates that 1,25-D3 can cross the blood–brain barrier (BBB) and alleviate ischemic stroke [7–9]. Consequently, we are leveraging the capacity of 1,25-D3 to traverse the BBB in vivo to explore its neuroprotective and endothelial repair mechanisms in diabetic brain injury.

Glucagon-like peptide-1 (GLP-1), an incretin produced by both brain and enteroendocrine cells, indirectly regulates glucose homeostasis by promoting insulin secretion and inhibiting glucagon release. Its receptor, GLP-1R, a G protein-coupled receptor, is widely expressed in neurons across the hypothalamus, hindbrain, and amygdala within the central nervous system, where it mediates the biological functions of GLP-1 upon activation. Extensive research has shown that GLP-1R activation has anti-inflammatory effects in multiple organs, including the heart, adipose tissue, retina, and kidneys, suggesting its potential to modulate neuroinflammation within the central nervous system [10–13]. Furthermore, activating GLP-1R not only mitigates inflammation to prevent neuronal apoptosis but also supports endothelial protection and repair [14]. GLP-1R influences gene transcription through pathways mediated by cyclic adenosine monophosphate-dependent protein kinase A (cAMP/PKA), extracellular signal-regulated kinase 1/2 (ERK1/2), and phosphatidylinositol 3-kinase/protein kinase B (PI3K/AKT) [15]. Notably, the PI3K/AKT pathway is critical for neuronal survival and endothelial repair, and its activation has been shown to protect neuronal and endothelial cells across various types of brain injury. Studies on experimental models of ischemic stroke and subarachnoid hemorrhage indicate that GLP-1R agonists and analogs can promote the phosphorylation of PI3K and AKT, inhibit inflammatory responses, and thereby provide neuroprotective and vasculoprotective effects [16, 17].

In diabetic conditions, decreased levels of tetrahydrobiopterin lead to endothelial nitric oxide (NO) synthase (e-NOS) uncoupling, resulting in endothelial dysfunction [18]. GLP-1 receptor agonists can interact with e-NOS to increase its expression in endothelial cells and catalyze the production of NO, a key vasodilator, thereby enhancing endothelial function. Vascular endothelial growth factor (VEGF) plays a crucial role in endothelial cell stabilization, angiogenesis, and neovascularization [3]. Recent studies have shown that VEGF exerts vascular-protective effects via e-NOS activation through the PI3K/AKT signaling pathway [19].

Streptozotocin (STZ) is a nitrosourea-derived Streptomyces acromogenes that causes pancreatic islet *β*-cell destruction and is used experimentally to produce a model of diabetes mellitus (DM) in mice and rats. However, the STZ-induced diabetic model has limitations, particularly in its representation of human diabetes, the chronicity of disease progression, and the complex neuroinflammatory responses. However, It has been invaluable for advancing our understanding of the mechanisms underlying diabetic complications [20, 21]. Studies have shown that 1,25-D3 could prevent renal oxidative damage, attenuate diabetic cardiac autophagy and damage in STZ-induced diabetes [22, 23], and GLP-1RA exhibits kidney-protective actions through understood oxidative stress and inflammation, autophagy mechanisms [24, 25].

Based on these, we hypothesize that GLP-1R and VEGF modulate e-NOS through the PI3K/AKT pathway, enhancing endothelial function and promoting vascular endothelium regeneration. This mechanism may offer therapeutic potential for addressing diabetic neurovascular damage. We have established a diabetic model using STZ and, by integrating molecular docking techniques with animal experiments, aim to preliminarily validate the protective effects of 1,25-D3 on diabetic brain injury. This protective role may be mediated through activation of the GLP-1R/VEGF-PI3K-AKT signaling pathway.

## 2. Materials and Methods

### 2.1. Molecular Docking

We docked 1,25-D3 with the related targets; specifically, first of all, we extracted the 2D structure of 1,25-D3 from the PubChem database, and then imported to Chem 3D software and exported into three-dimensional conformation, and through the software's MM calculation to minimize the energy, so as to optimize the structure to reserve. Then, we selected the three-dimensional crystal structure of the target protein from the PDB protein database, and the PDBID was GLP-1R (6GB1), PI3K (4JPS), AKT1 (3CQW), e-NOS (3EAH), intercellular adhesion molecule-1 (ICAM-1) (1P53), vascular intercellular adhesion molecule-1 (VCAM-1) (1IJ9), and VEGF-*α* (4WPB), respectively. We imported the above target proteins into PyMOL1.7.2.1 to remove solvent and remove organic, exported pdb files into AutoDockTools1.5.6 to bind hydrogenation, and exported them as pbdqt files. At the same time, 1,25-D3 was imported into AutoDockTools1.5.6 software as a ligand, and pdbqt file was exported. The Grid option was used to determine the active site of molecular docking and set the size. Finally, AutodockVinav.1.1.2 was run for molecular docking, respectively, the binding energy was calculated, and the visualization was realized by PyMOL1.7.2.1 software.

### 2.2. Animals

We recruited 8-week-old specific pathogen-free (SPF) male Sprague-Dawley (SD) rats (weight, 280–300 g) from the Weifang Medical University Experimental Animal Center and kept them in a cage with a regulated temperature on a 12-h/12-h light/dark cycle. All rats had free access to water and food. Our animal treatments were carried out in accordance with the National Institutes of Health's (NIH) Guide for the Care and Use of Laboratory Animals. The Ethics Committee for the Use of Experimental Animals at Shandong Second Medical University approved all animal procedures (application approval number is 2018-037).

### 2.3. Models

We treated SD rats with a high-fat diet (HFD: 60% energy from fat) combined with low-dose STZ (HFD/STZ) to establish a Type 2 diabetic model. The SD rats (*n* = 40) were randomly divided into normal control (NC) group (*n* = 10), untreated diabetic (DM) group (*n* = 10), diabetes plus 1,25-D3 (DM + 1,25-D3) treatment group, and diabetes plus 1,25-D3 and VDR antagonist (P5P, pyridoxal-5-phosphate) (DM + 1,25-D3 + P5P) group (*n* = 10). The NC group rats were provided a normal chow diet (NCD), and the diabetic model group accepted the HFD for 8 weeks. After 8 weeks' HFD, a single dose of 35 mg/kg STZ (Sigma–Aldrich, Saint Louis, MO, USA) dissolved in 2% citrate buffer was intraperitoneally injected into HFD-fed rats once a week for 3 weeks. The SD rats, which were confirmed by fasting plasma glucose of more than 16.7 mmol/L, were included into the diabetic model. After creating the Type 2 diabetic model, the 1,25-D3 and 1,25-D3 + P5P groups were intraperitoneally injected with 1,25-D3 and 1,25-D3 + P5P once a day for 3 consecutive days. The rats received 5 µg/kg 1,25-D3 (Sigma–Aldrich, St. Louis, MO, USA) by intraperitoneal injection in 1% dimethyl sulfoxide (DMSO, Sigma–Aldrich, St. Louis, MO, USA) and P5P (Sigma–Aldrich, St. Louis., MO, USA) was dissolved in 0.9% NaCl and administered intraperitoneally at a dose of 0.4 mg/kg. As a control group, rats received vehicle injections (sodium citrate buffer, 2%) by intraperitoneal injection once a week for 3 weeks. The body weight and plasma blood glucose levels of rats were monitored. Then, the rats were sacrificed after the last injection.

### 2.4. Hematoxylin and Eosin (H&E) Staining

Four percent paraformaldehyde was used to fix the brain tissue samples. The 5-m paraffin slices were stained with H&E after deparaffinization and rehydration. The histopathological changes in the brain were observed under the light microscope.

### 2.5. Western Blot (WB) Analysis

The rats were euthanized after being sedated and placed on a dissection table. Frozen sterile saline was injected through the heart; then, the brain cortex tissue was extracted, detached, and stored in a −80°C refrigerator. Total protein was obtained from the supernatant by placing the brain cortex tissue in RIPA lysis buffer containing protease and phosphatase inhibitors and centrifuging at 14,000 rpm for 20 min at 4°C. Protein concentration was determined using a BCA assay kit (Beyotime, China). Then, equal amounts of protein (30 µg/lane) were separated by SDS–PAGE and transferred to PVDF (Millipore, USA) membranes. Nonspecific binding was blocked with TBST containing 5% skimmed milk for 2 h, and the membranes were then incubated with rabbit anti-GLP1-R (1:1000, Abcam, ab21853, USA), anti-VEGF-*α* (1:1000, Abcam, USA), anti-PI3K (1:1000, Abcam, ab191606, USA), anti-p-PI3K (1:750, Abcam, USA), anti-AKT (1:10000, Abcam, China), anti-p-AKT (1:1000, Abcam, USA), anti-*β*-actin (1:5000, Protein Tech Group, China) at 4°C overnight. The membranes were treated with the HRP-conjugated anti-rabbit IgG secondary detection antibody (1:5000, Beyotime, China) for 1 h at room temperature after three 10-min washes in TBST. Protein bands were visualized with enhanced chemiluminescence using an ECL kit (Beyotime, Shanghai, China). Analysis of specific bands was performed using ImageJ software (NIH, Bethesda, MD, USA) and was standardized to that of *β*-actin.

### 2.6. Quantitative Real-Time PCR

Each rat's brain cortex was detached and placed in an enzyme-free tube that had been prepared ahead of time. Total RNA was isolated from brain tissue using Trizol reagent (Thermo Fisher, USA), and cDNA was reverse transcribed using the HiFi Script cDNA Synthesis Kit (CWBIO, Taizhou, China) at 45°C for 15 min and 85°C for 5 min. The samples were briefly centrifuged and chilled on ice at the end of the procedure. Following that, the target gene fragment was amplified using the Ultra SYBR Mixture (CWBIO, Taizhou, China). The following were the real-time PCR reaction conditions: 95°C predenaturation for 10 min, denaturation at 95°C for 15 s, annealing and extension at 60°C for 1 min, for a total of 39 cycles. The gene's relative mRNA level was determined using the 2^−*ΔΔ*^CT technique. As a control gene, *β*-actin was employed.  Table 1 shows the sequences of primers.

### 2.7. Enzyme-Linked Immunosorbent Assay (ELISA)

The brain cortex tissue was homogenized in PBS buffer and centrifuged at 3000 rpm at 4°C for 20 min to obtain total protein from the supernatant. The levels of ICAM-1 and VCAM-1 were assayed according to the ELISA kits (Meimian, Jiangsu, China), and the level of e-NOS was measured using e-NOS ELISA kits (MLBIO, Shanghai, China) following the manufacturer's instructions. The optical density (OD) was detected at 450 nm.

### 2.8. Statistical Analysis

The statistical analysis was performed with GraphPad Prism 9 (Graph Pad Software Inc, San Diego, CA, USA). All obtained data are presented as the mean ± SEM. The comparisons between multiple groups were analyzed by one-way ANOVA, and Tukey's post hoc test was used for further pairwise comparisons. A *p* value < 0.05 was considered statistically significant.

## 3. Results

### 3.1. Analysis of Binding Ability of 1,25-D3 and Related Targets

**Figure 1:**
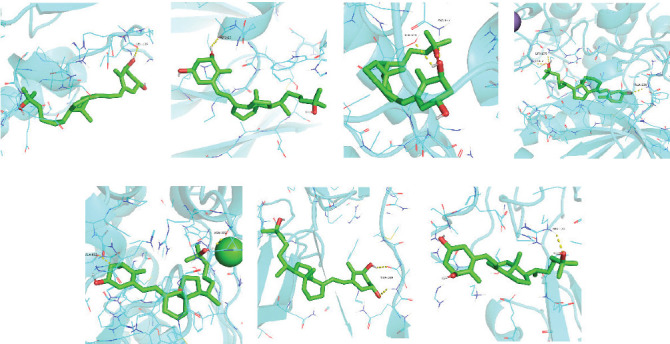
Visualization of molecular docking results. (A) 1,25-D3 + GLP-1-R. (B) 1,25-D3 + VEGF-*α*. (C) 1,25-D3 + PI3K. (D) 1,25-D3 + AKT. (E) 1,25-D3 + e-NOS. (F) 1,25-D3 + ICAM-1. (G) 1,25-D3 + VCAM-1. 1,25-D3, 1*α*,25-dihydroxyvitamin D3; e-NOS, endothelial nitric oxide synthase; GLP-1, glucagon-like peptide-1; ICAM-1, intercellular adhesion molecule-1; VCAM-1, vascular intercellular adhesion molecule-1; VEGF-*α*, vascular endothelial growth factor-*α*.

Seven protein targets with binding energy ≤−5 kcal/mol were obtained by molecular docking, indicating that 1,25-D3 had good affinity with related proteins (all molecular docking results are shown in Supporting Information 1: Figure S1). Specifically, 1,25-D3 and GLP-1R exhibit strong binding (−6.3 kcal/mol), as shown in Table 2 and in Figure 1, and hydrogen bonds are formed between 1,25-D3 and LEU-118 of GLP-1R when evaluated from the interaction point. 1,25-D3 also forms a hydrogen bond with VEGF-*α* to achieve tight binding (−6.1 kcal/mol), and the interaction site is to form a hydrogen bond with ARG-23 on VEGF-*α*. 1,25-D3 also strongly binds to PI3K (−8.4 kcal/mol), and hydrogen bonds are formed between 1,25-D3 and THR-679 and ASN-677 of PI3K. Similarly, 1,25-D3 exhibits strong binding to AKT1 (−8.5 kcal/mol) and is hydrogen bonded to SER-7, LYS-276, and ALA-230 of AKT1. 1,25-D3 forms hydrogen bonds with ASN-332 and ALA-412 of e-NOS, and has strong bonding ability (−8.5kcal/mol). 1,25-D3 also acts on THR-389 at the ICAM-1 action site to bind with two hydrogen bonds, and the binding ability is strong (−6.8 kcal/mol). In addition, 1,25-D3 and ARG-123 of VCAM-1 are linked by hydrogen bonds, forming a strong bond (−5.9 kcal/mol). In summary, the binding energy of all the targets was lower than −5.0 kcal/mol, suggesting that all of them had good binding activity with 1,25-D3, and there were three active groups with strong binding activity less than −7.0 kcal/mol.

### 3.2. Blood Glucose

**Figure 2:**
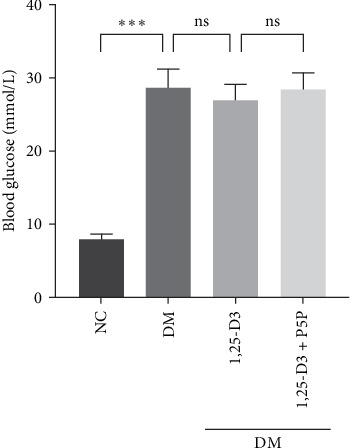
Effects of 1,25-D3 on blood glucose in STZ-induced diabetic rat brains. All experiments were repeated at least three times (*n* = 10, each group). *⁣*^*∗*^*p* < 0.05, *⁣*^*∗∗*^*p* < 0.01. 1,25-D3, 1*α*,25-dihydroxyvitamin D3; H&E, hematoxylin and eosin; P5P, pyridoxal-5-phosphate, antagonist of VDR; STZ, streptozotocin.

To confirm the effect of 1,25-D3 on plasma blood glucose in rats, we analyzed the blood glucose levels of rats in each group. The results are shown in Table 3. Comparing with the NC group, the blood glucose level in the DM group was significantly increased. Blood glucose levels were lower in the 1,25-D3 group compared to the DM group. The 1,25-D3 + P5P group was higher than the 1,25-D3 group (Figure 2). There was no significant difference in blood glucose levels between DM group and 1, 25-D3 group, between DM group and 1, 25-D3 +P5P group, between 1, 25-D3 group and 1, 25-D3 +P5P group. There was no statistically significant difference in blood glucose levels among the DM group, 1, 25-D3 group and 1, 25-D3 +P5P group.

### 3.3. 1,25-D3 Inhibited Hyperglycemia-Induced Brain Damage in Rats

**Figure 3:**
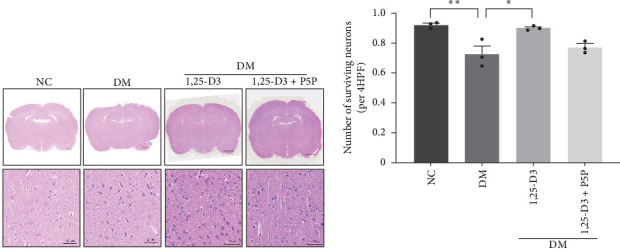
Effects of 1,25-D3 on pathology in STZ-induced diabetic rat brains. (A) Pathological changes in the intact brain of rats through H&E (×1). The selection of a white box represents the enlarged area corresponding to the image below. NC and DM group (scale bar: 1000 μm), 1,25-D3 and 1,25-D3 + P5P group (scale bar: 1600 μm); pathological changes of the brain cortex rat were identified by H&E (×40). NC, DM, 1,25-D3, and 1,25-D3 + P5P group (scale bar: 50 μm). (B) Quantification of H&E staining. All experiments were repeated at least three times (*n* = 3, each group). *⁣*^*∗*^*p* < 0.05, *⁣*^*∗∗*^*p* < 0.01. 1,25-D3, 1*α*,25-dihydroxyvitamin D3; DM, diabetes mellitus; H&E, hematoxylin and eosin; NC, normal control; P5P, pyridoxal-5-phosphate, antagonist of VDR; STZ, streptozotocin.

H&E staining was performed to observe the morphological and histological changes in the cortical region of rats, and the results were analyzed.  Figure 3A depicts the results obtained using a light microscope (all images can be found in Supporting Information 2: Figure S2). Normal rats' neurons possessed a complete and cleanly ordered structure, normal cell shape, and discernible nuclei. Compared with the normal group, the structure of neurons in the rat brain tissue of the diabetic group was incomplete, the cells were swollen, and the nuclei were pyknotic; the number of surviving neurons increased significantly; additionally, diabetic rats treated with 1,25-D3 had the structural integrity of neurons, cytoplasmic vacuolization was reduced, and the degree of cell swelling and nuclear pyknosis was reduced compared with non-treated diabetic counterparts. In addition, it can be seen from Figure 3B that the number of neuronal cell death was significantly reduced compared with that in the diabetes group. Then, the changes induced by 1,25-D3 were reversed in some cases in the inhibitor group. These indicated that 1,25-D3 alleviates brain injury induced by diabetes.

### 3.4. 1,25-D3-Regulated Endothelial Function and Angiogenesis in STZ-Induced Diabetic Rats

**Figure 4:**
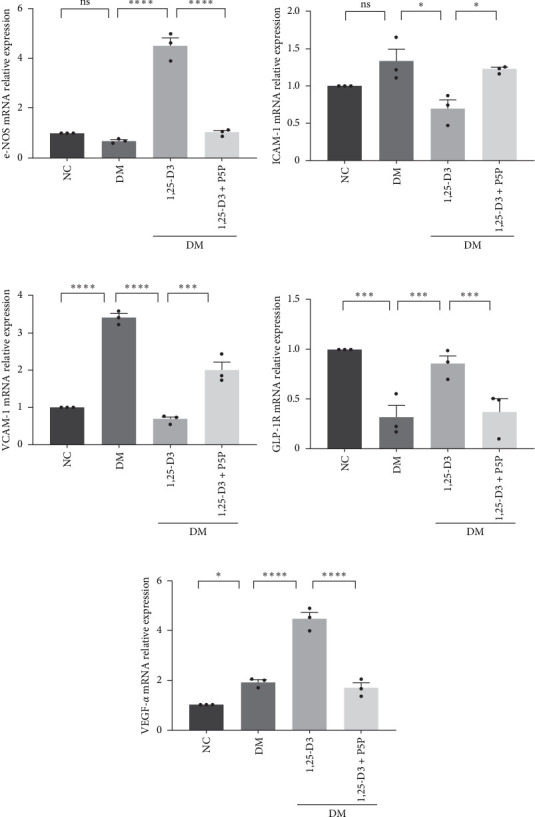
Changes in the expression of relevant mRNA in diabetic rats induced by STZ. The mRNA expression of e-NOS (A), ICAM-1 (B), VCAM-1 (C), GLP-1R (D), VEGF-*α* (E). Data were represented as mean ± SEM. All experiments were repeated at least three times (*n* = 3, each group). *⁣*^*∗*^*p*  < 0.05, *⁣*^*∗∗*^*p*  < 0.01, *⁣*^*∗∗∗*^*p*  < 0.001, *⁣*^*∗∗∗∗*^*p*  < 0.0001. 1,25-D3, 1*α*,25-dihydroxyvitamin D3; e-NOS, endothelial nitric oxide synthase; GLP-1, glucagon-like peptide-1; ICAM-1, intercellular adhesion molecule-1; P5P, pyridoxal-5-phosphate, antagonist of VDR; STZ, streptozotocin; VCAM-1, vascular intercellular adhesion molecule-1; VEGF-*α*, vascular endothelial growth factor-*α*.

**Figure 5:**
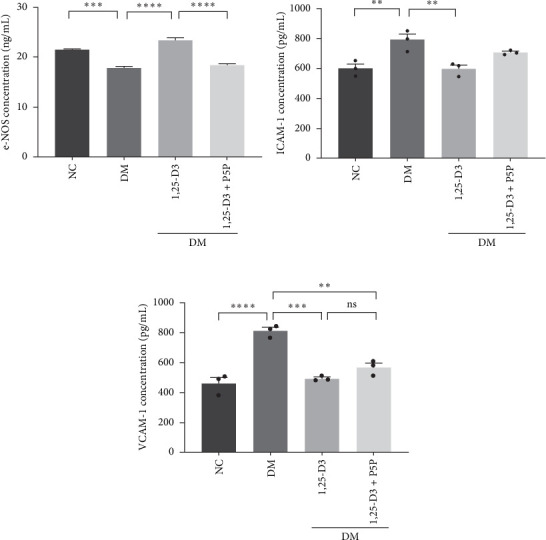
Changes in the expression of relevant proteins in diabetic rats induced by STZ. Quantification of e-NOS (A), ICAM-1 (B), VCAM-1 (C). Data were represented as mean ± SEM. All experiments were repeated at least three times (*n* = 3, each group). *⁣*^*∗*^*p*  < 0.05, *⁣*^*∗∗*^*p*  < 0.01, *⁣*^*∗∗∗*^*p*  < 0.001, *⁣*^*∗∗∗∗*^*p*  < 0.0001. 1,25-D3, 1*α*,25-dihydroxyvitamin D3; e-NOS, endothelial nitric oxide synthase; ICAM-1, intercellular adhesion molecule-1; P5P, pyridoxal-5-phosphate, antagonist of VDR; STZ, streptozotocin; VCAM-1, vascular intercellular adhesion molecule-1.

**Figure 6:**
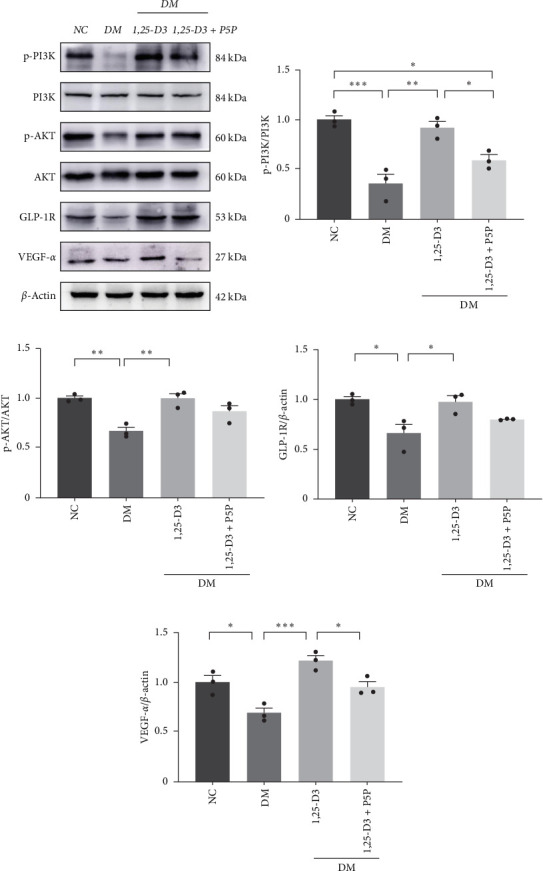
1,25-D3 promoted the expression of GLP-1R, AKT, p-AKT, p-PI3K, p-PI3K, and VEGF-*α* in diabetic rats induced by STZ. Western blot showed the expression of GLP-1R, AKT, p-AKT, p-PI3K, p-PI3K, and VEGF-*α* in the brain cortex (A). Quantification of p-AKT (B), p-PI3K (C), GLP-1R (D), VEGF-*α* (E) in (A). Data were represented as mean ± SEM. All experiments were repeated at least three times (*n* = 3, each group). *⁣*^*∗*^*p*  < 0.05, *⁣*^*∗∗*^*p*  < 0.01, *⁣*^*∗∗∗*^*p*  < 0.001. 1,25-D3, 1*α*,25-dihydroxyvitamin D3; GLP-1, glucagon-like peptide-1; P5P, pyridoxal-5-phosphate, antagonist of VDR; STZ, streptozotocin; VEGF-*α*, vascular endothelial growth factor-*α*.

By examining the mRNA levels and protein expression of e-NOS and VEGF, we explored the effect of 1,25-D3 on vascular endothelial function (Supporting Information 3: Table S1, Supporting Information 4: Table S2, Supporting Information 5: Figure S3, Supporting Information 6: Figure S4, Supporting Information 7: Figure S5, Supporting Information 8: Figure and S6). The experimental results showed that compared with the normal group, the contents of e-NOS and VEGF in the brain tissue of the diabetes group were significantly reduced, and the levels of e-NOS, VEGF mRNA, and protein expression in the 1,25-D3 treatment group were significantly higher than those in the diabetes group. Specifically, after the addition to inhibitors, the levels of e-NOS and VEGF were significantly decreased and the positive effects of 1,25-D3 were suppressed. The results are shown in Figures 4A,E, 5A, and 6E.

### 3.5. 1,25-D3 Reduced the Production of Pro-Inflammatory Cytokines

To investigate the effect of 1,25-D3 on the inflammatory response of brain tissue in STZ-induced diabetic rats, we used quantitative RT-PCR and ELISA to quantify the mRNA and protein levels of inflammatory markers ICAM-1 and VCAM-1, respectively (Supporting Information 3: Table S1 and Supporting Information 4: Table S2). The quantitative RT-PCR results revealed that, when compared to the normal group, the mRNA levels of ICAM-1 and VCAM-1 in the brain tissue of the model group rats were significantly increased; however, after 1,25-D3 intervention, the mRNA levels of ICAM-1 and VCAM-1 were significantly reduced as compared to the model group. P5P, on the other hand, counteracted the positive effects of 1,25-D3 (Figure 4B,C). ELISA's findings were consistent with the quantitative RT-PCR results in that the levels of ICAM-1 and VCAM-1 in diabetes rats were much greater than in normal rats, but the treatment of 1,25-D3 reduced the expression levels of ICAM-1 and VCAM-1. The protective effect of 1,25-D3 was also eliminated by the inhibitor (Figure 5B,C).

### 3.6. 1,25-D3 Activated GLP-1R to Attenuate Neuroinflammation Through PI3K/AKT Pathway

To elucidate the vascular protective mechanism and anti-inflammatory effect of 1,25-D3 on hyperglycemic brain injury, we determined the expression levels of GLP-1R, PI3K, p-PI3K, AKT, and p-AKT proteins in brain tissue of rats pretreated with 1,25-D3 and protein pathway inhibitors, as well as GLP-1R mRNA transcription levels (Figures 4D, 6A, Supporting Information 3: Table S1, Supporting Information 4: Table S2, Supporting Information 5: Figure S3, Supporting Information 6: Figure S4, Supporting Information 7: Figure S5, and Supporting Information 8: Figure S6). It is shown in Figure 6D that 1,25-D3 can activate the GLP-1 receptor in STZ-induced diabetic rats, whereas 1,25-D3 antagonists can inhibit GLP-1R transcription and protein expression. Similarly, WB was used to assess the phosphorylation forms of PI3K and AKT. Diabetic rats' brain tissue had lower levels of p-PI3K and p-AKT protein expression than the control group, but diabetic rats treated with 1,25-D3 had higher levels of protein expression. Furthermore, when 1,25-D3 action is inhibited, the upregulation of PI3K and AKT phosphorylation levels by 1,25-D3 is hindered (Figure 6B,C). According to the research presented above, 1,25-D3 may be able to increase GLP-1R/PI3K/AKT signaling, resulting in protective vascular and anti-neuroinflammatory actions.

## 4. Discussion

Diabetes is an ongoing disorder that causes hyperglycemia and chronic inflammation in the body, which can lead to endothelial dysfunction. Long-term chronic glucose metabolism disorders can cause brain tissue damage, resulting in pathological changes in brain structure, neurophysiology, and other aspects, as well as increased levels of inflammatory factors and decreased levels of vascular protective factors. Recent research has demonstrated that some functional parameters, such as anti-inflammatory and anti-endothelial dysfunction, protect the structure of the cerebral cortex and the number of nerve cells, delaying the onset of diabetes encephalopathy.

Neuroinflammation is frequently regarded as the primary cause of neurological disorders. Neuroprotective advantages conferred by 1,25-D3 have been documented for ischemic brain injury, Parkinson's disease, Alzheimer's disease, and other neurodegenerative illnesses through modulating autophagy, neuroinflammation, cell apoptosis, oxidative stress, and endothelial cell damage [26–29]. These investigations, however, have not established the mechanism involved in the effect of 1,25-D3 on diabetes-related brain damage. Based on previous research, we used molecular docking and animal experiments to investigate the effect of 1,25-D3 on STZ-induced brain injury in diabetic rats and its possible molecular mechanism. First, we performed molecular docking, and the results showed that 1,25-D3 had a good binding activity with seven possible targets (GLP-1R, PI3K, AKT1, VEGF-*α*, e-NOS, ICAM-1, and VCAM-1). There were three active targets with strong binding activity (less than −7.0 kcal/mol), suggesting that 1,25-D3 can strongly bind to these action targets and exert its neuroprotective effect. Second, H&E staining conveys direct pathological evidence that 1,25-D3 can alleviate brain cortex structural damage after diabetes. We also established that 1,25-D3 dramatically increased e-NOS and VEGF-*α* expression in diabetic rats while suppressing adhesion inflammatory factors and improving vascular endothelial function. Furthermore, 1,25-D3 therapy drastically boosted GLP-1R expression and PI3K/AKT activation in cortical neurons after diabetic brain damage. Third, our experiment results confirmed that the levels of blood glucose of the 1,25-D3 group, diabetes group, and 1,25-D3 + P5P group, and the three groups showed no significant difference. This implicated that 1,25-D3 did not affect blood glucose. 1,25-D3 may not be a primary agent for lowering blood glucose in diabetic individuals. Its inhibitive effects on diabetic brain injury are not likely through lowering blood glucose or decreasing advanced glycosylation end products. Instead, its role appears to be more modulatory, improving insulin sensitivity and influencing inflammation and cellular function. Finally, P5P reversed the beneficial impact of 1,25-D3 on neurological injury and endothelial dysfunction by modifying the expression of inflammatory and vascular protection factor-associated proteins such as GLP-1R, VEGF-*α*, e-NOS, ICAM-1, and VCAM-1. The results of this research suggest that 1,25-D3 is significant in declining neuroinflammation and enhancing vascular endothelial function following brain injury in diabetic rats through GLP-1R/PI3K/Akt signaling pathway.

GLP-1 is a naturally occurring small molecule peptide that promotes insulin release in hyperglycemia. It is produced in the small intestine by L cells and is commonly used to treat diabetes and its consequences. GLP-1R is a seven-transmembrane domain G protein-coupled receptor that is activated by GLP-1 and has a biological role. GLP-1R agonists have been shown to decrease stomach emptying, block glucagon secretion, and promote insulin secretion in the regulation of blood glucose homeostasis [30]. Furthermore, Puddu and Maggi [11] demonstrated that GLP-1R protects the blood-retinal barrier, weakens vascular permeability, and inhibits neuronal apoptosis to protect neural function in diabetes retinopathy [11, 12, 16, 31]. GLP-1R stimulation improves acute brain injury after subarachnoid hemorrhage by reducing neuronal damage and inflammation [16, 17]. Moreover, stimulation of GLP-1R may widen cerebral arteries, increase blood flow, reduce infarct volume, and ameliorate neurological deficit symptoms produced by ischemic stroke [28], which implies that GLP-1R agonists have neurotrophic and neuroprotective effects in nervous system disease [30]. However, natural endogenous GLP-1 has a half-life of only a few minutes and is easily degraded by the enzyme dipeptidylpeptidase-4 (DPP-4), exhibiting an extremely low affinity for classical GLP-1R. Therefore, the repair effects of exogenous long-acting GLP-1 analogs or GLP-1R agonist analogs on nerves and blood vessels are attracting increased interest [32]. In our study, we tested protein imprinting and PCR to detect the mRNA and protein levels of GLP-1R in the cerebral cortex of diabetes rats. The findings revealed that GLP-1R protein expression in diabetes rats was reduced, and 1,25-D3 reversed this change, which could activate GLP-1R to achieve the neuroprotective effect.

As stated previously, the PI3K/AKT pathway is a well-known route linked to neuronal survival in various models of nervous system disorders [13, 24, 25]. Upregulated p-AKT has been identified to be regulated by downstream targets such as e-NOS, GSK-3, mTOR, and NF-k B to mitigate neuronal damage [16, 33–36]. Endothelial cells secrete vasoconstrictor and inflammatory substances in response to long-term hyperglycemia, lowering NO production and leading to vascular fragility and impaired endothelial repair [37]. Activating the PI3K/AKT/e-NOS pathway via the GLP-1R has been shown to regulate microvascular dilation and contraction function, prevent endothelial cells from transforming into mesenchymal cells following STZ-induced vascular injury, and inhibit the secretion of pro-inflammatory cytokines [12, 31]. Furthermore, 1,25-D3 has been shown to boost VEGF expression and mediate neovascularization in a rat model of cerebral ischemia-reperfusion [3]. AKT plays a key role in several cardiovascular functions, such as angiogenesis. In this study, compared with the DM group, the PI3K/AKT signaling pathway was activated, and the VEGF expression level increased in the DM + 1,25-D3 group, which may be the result of AKT induction to promote angiogenesis. VEGF can activate the PI3K/AKT/e-NOS pathway in human umbilical vein endothelial cells (HUVECs) treated with high glucose, thereby enhancing the survival and migration of pancreatic microvascular endothelial cells, hence avoiding the effects of hyperglycemia [37]. These findings are consistent with our studies, which show that 1,25-D3 can activate the GLP-1R/VEGF-PI3K/AKT signaling pathway, increasing e-NOS levels, accelerating NO bioavailability, mediating vascular protection, and improving endothelial dysfunction.

Cell adhesion molecule (CAM) interaction is critical in mediating immunological and inflammatory responses [38]. Intercellular adhesion molecule-1 (ICAM-1) and vascular intercellular adhesion molecule-1 (VCAM-1) are two proteins that facilitate the adherence of circulating leukocytes to endothelial cells and then infiltrate extravascular brain parenchyma, causing inflammation after brain damage. ICAM-1 and VCAM-1 are intermittently expressed in endothelial cells and smooth muscle cells in normal arteries, with a high density in plaque-prone locations, and play a significant role in atherosclerosis and endothelial dysfunction [39, 40]. ICAM-1 and VCAM-1 are both important molecules in hyperglycemic brain injury produced by vascular instability, whether in terms of stimulating leukocyte adhesion, aggregation, or chemotaxis to cause vasculitis or the formation of cerebral atherosclerotic plaque [41]. Our findings further demonstrate that in high glucose situations, excessive levels of ICAM-1 and VCAM-1 promote vascular dysfunction, whereas 1,25-D3 can downregulate adhesion factors, reduce inflammation, and further enhance vascular function. Endothelial cells enhanced the expression of ICAM-1 and VCAM-1 in the experimental colitis model caused by trinitrobenzene sulfonic acid, increasing leukocyte recruitment to the inflammatory bowel, which was consistent with our result [42].

Of course, our research still has some limitations. To begin, our study only included in vivo studies, and the mechanism of action in vitro requires more investigation. Previous research has demonstrated that 1,25-D3 might cross the BBB and bind to these proteins, exerting biological effects. However, we only looked at the effects of vascular endothelial relaxation factors and inflammatory factors on vascular endothelial dysfunction and failed to reveal any effects of 1,25-D3 on BBB leakage, permeability, or expression of endothelial tight junction-related molecules such as ZO-1, occludin, and claudin-5 [6–9]. As a result, more research into the relevant signal pathways and molecular processes of 1,25-D3 on brain damage in diabetes is required. Furthermore, although the effect of 1,25-D3 on intracellular neuronal glucose is increasingly receiving attention and controversy, limited by the technical conditions, we were not able to measure intraneural glucose concentration before and after 1,25-D3 injection.

## 5. Conclusions

Our research results indicate that 1,25-D3 can strongly bind to signaling molecules in the GLP-1R/PI3K/AKT pathway, thereby activating the pathway to improve the protective effect, as evidenced by increased levels of vascular protective factors, as well as decreased levels of inflammatory factors. These findings suggest that inflammation and vascular dysfunction play important roles in the etiology of diabetic brain injury, and 1,25-D3 supplementation provides preclinical evidence for the future prevention and treatment of hyperglycemic brain injury, making it a possible therapy option. Of course, much remains to be validated through further pharmacokinetic and CNS targeting studies about 1,25-D3.

## Figures and Tables

**Table 1:** Primers used for quantitative real-time polymerase chain reaction.

Target gene	Primer sequence
GLP-1R	Forward: 5′ – TCCTTCATCCTCCGAGCACTGTC - 3′ Reverse: 5′ – GCCCAGAGAGTCCTGATACGAGAG - 3′

VEGF-*α*	Forward: 5′- CCGTCCTGTGTGCCCCTAATG - 3′ Reverse: 5′ - CGCATGATCTGCATAGTGACGTTG - 3′

e-NOS	Forward: 5′ - GCCACCTGATCCTAACTTGCCTTG - 3′ Reverse: 5′ -TCGTGTAATCGGTCTTGCCAGAATC - 3′

ICAM-1	Forward: 5′ - ACTATCGAGTGGACACAACTGG - 3′ Reverse: 5′ - TGCCACAGTTCTCAAAGCAC - 3′

VCAM-1	Forward: 5′-GTGTGAAGGAGTGAATCTGGTTGGG- 3′ Reverse: 5′ - CTCAGCGTCAGTGTGGATGTAGC - 3′

Abbreviations: e-NOS, endothelial nitric oxide synthase; GLP-1, glucagon-like peptide-1; ICAM-1, intercellular adhesion molecule-1; VCAM-1, vascular intercellular adhesion molecule-1; VEGF-*α*, vascular endothelial growth factor-*α*.

**Table 2:** Binding energy of molecular docking(kcal/mol).

Group	GLP-1R	VEGF-*α*	PI3K	AKT	e-NOS	ICAM-1	VCAM-1
1,25-D3	−6.3	−6.1	−8.4	−8.5	−8.5	−6.8	−5.9

Abbreviations: 1,25-D3, 1*α*,25-dihydroxyvitamin D3; e-NOS, endothelial nitric oxide synthase; GLP-1, glucagon-like peptide-1; ICAM-1, intercellular adhesion molecule-1; VCAM-1, vascular intercellular adhesion molecule-1; VEGF-*α*, vascular endothelial growth factor-*α*.

**Table 3:** Weight and blood glucose of all rats.

Testing index	NC	DM	1,25-D3	1,25-D3 + P5P
Weight (g)	274 ± 30	245 ± 25	236 ± 25	245 ± 23
Blood glucose (mmol/L)	7.84 ± 0.75	28.58 ± 2.61	26.86 ± 2.12	28.32 ± 2.34

*Note:* Weight values are expressed as “mean ± range,” and blood sugar values are expressed as “mean ± standard deviation.”

Abbreviations: 1,25-D3, 1*α*,25-dihydroxyvitamin D3; DM, diabetes mellitus; NC, normal control.

## Data Availability

The data used and analyzed in the study are available from the corresponding author upon reasonable request.

## Supporting Information

Supporting Information 1 Figure S1: images of molecular docking.

Supporting Information 2 Figure S2: images of H&E staining.

Supporting Information 3 Table S1: the CT value of PCR and actual concentrations.

Supporting Information 4 Table S2: the relative concentrations of mRNA.

Supporting Information 5 Figure S3: summary of WBs in all groups.

Supporting Information 6 Figure S4: images of WBs in group one.

Supporting Information 7 Figure S5: images of WBs in group two.

Supporting Information 8 Figure S6: images of WBs in group three.
