# Supplementary figures and images for: 1,25-D3 Protects Diabetic Brain Injury Through GLP-1R/PI3K/Akt Pathway by Experimental and Molecular Docking Studies
Source: Mediators Inflamm. 2025 Mar 7;2025:8217035. doi: 10.1155/mi/8217035 (PMC11986256; doi:10.1155/mi/8217035)

A

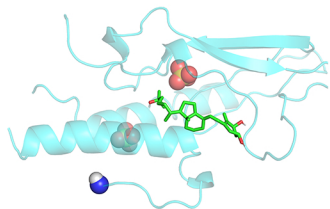

VitD3-GLP-1R

B

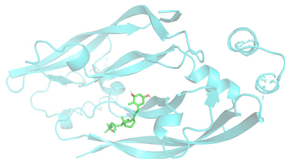VitD3-VEGF $\alpha$ 

C

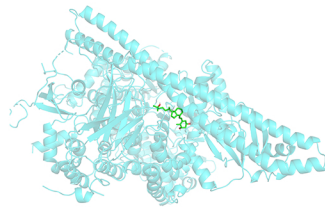

VitD3-PI3K

D

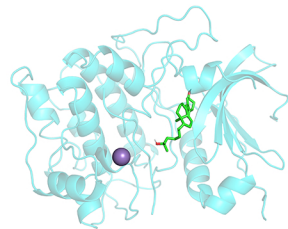

VitD3-AKT1

E

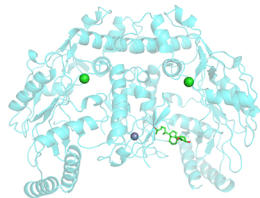

VitD3-e-NOS

F

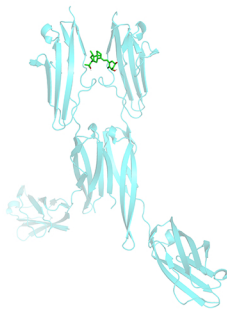

VitD3-ICAM-1

G

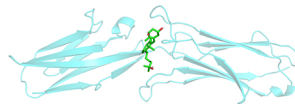

VitD3-VCAM-1

Supplement: Supporting Information 1 — Figure S1: images of molecular docking. [file 8217035.f1.pdf]

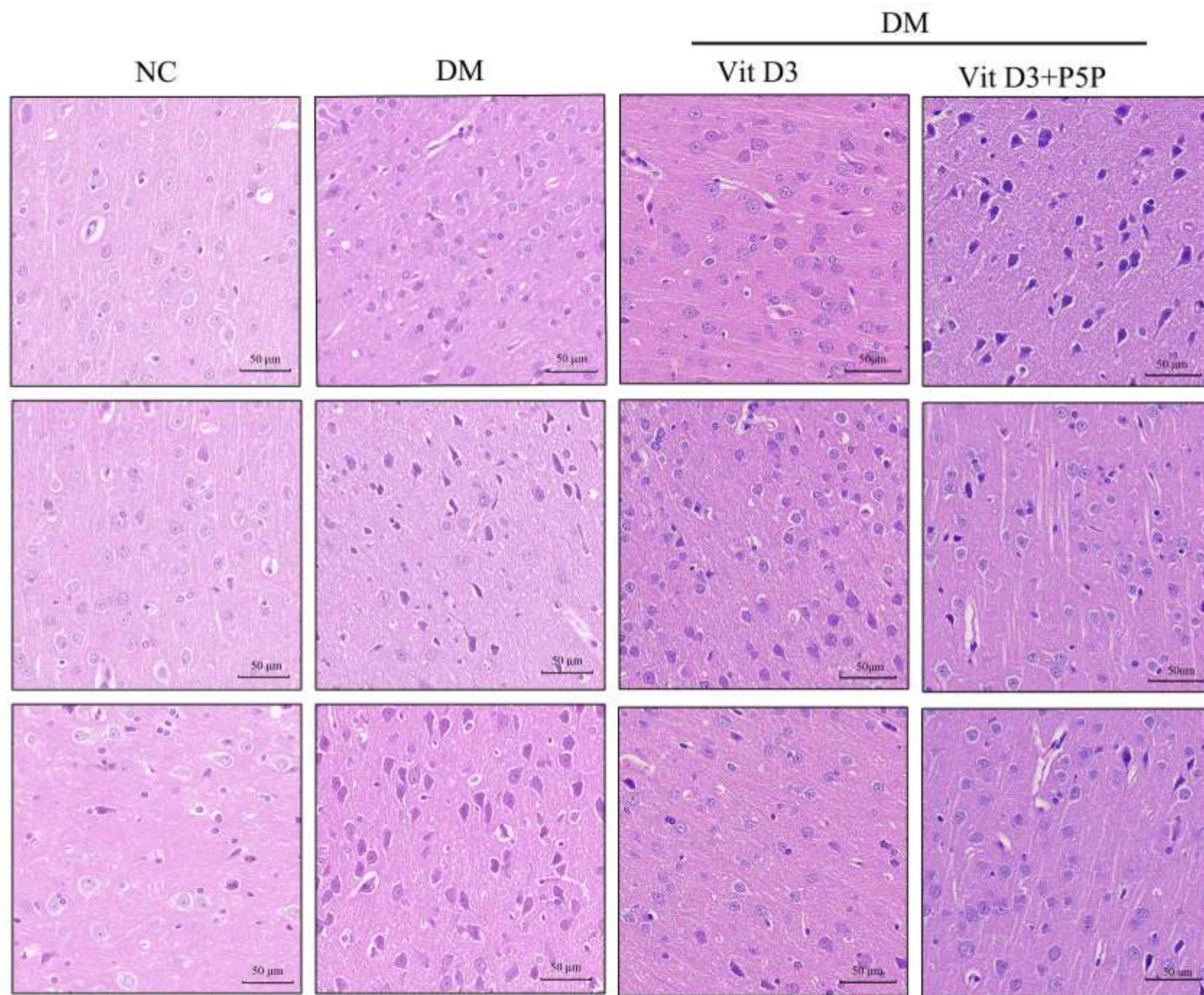

Supplement: Supporting Information 2 — Figure S2: images of H&E staining. [file 8217035.f2.pdf]

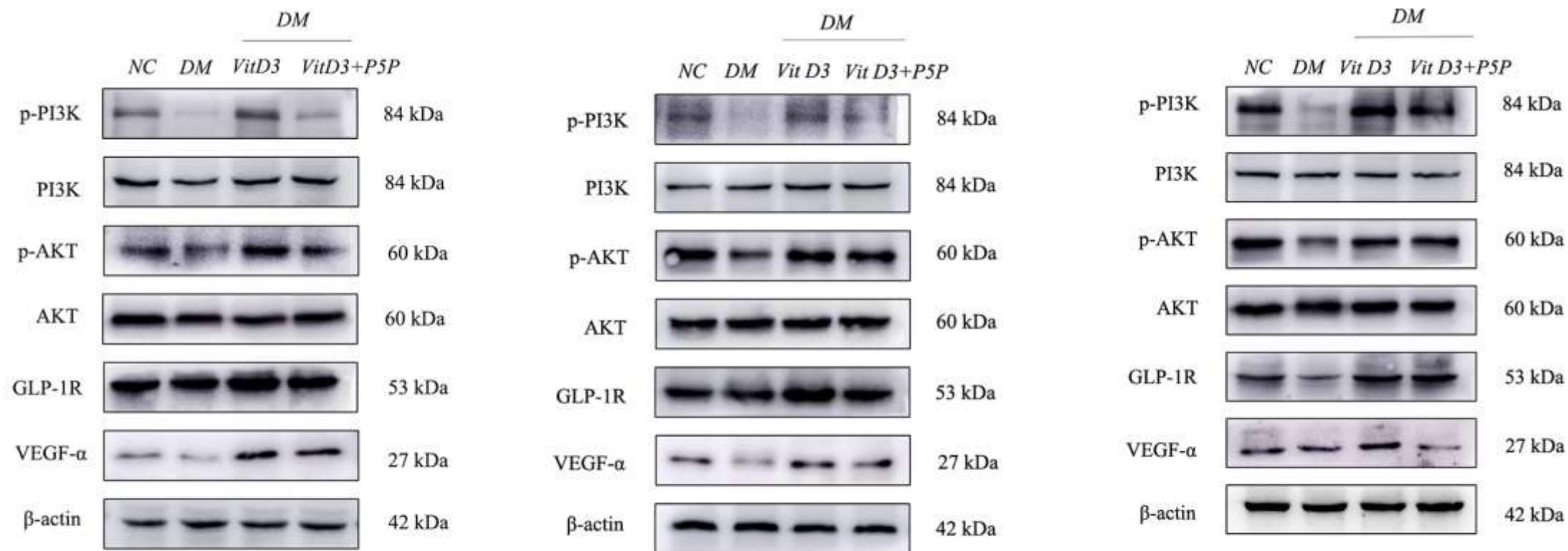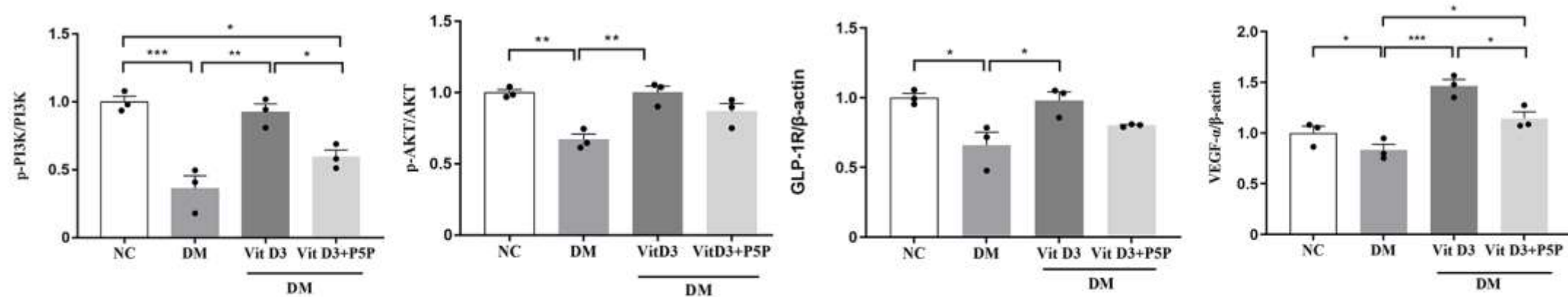

Supplement: Supporting Information 5 — Figure S3: summary of WBs in all groups. [file 8217035.f5.pdf]

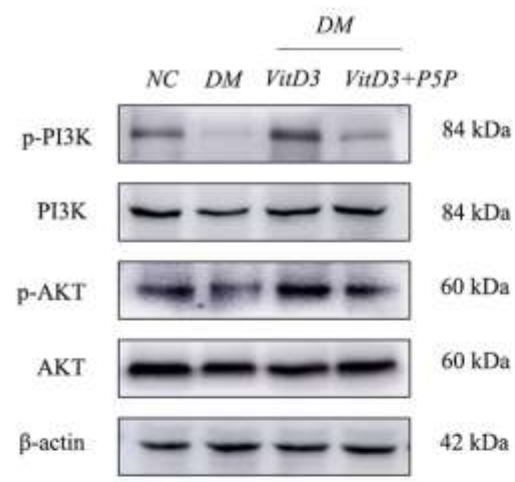

p-PI3K

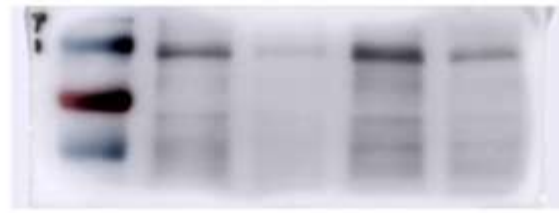

PI3K

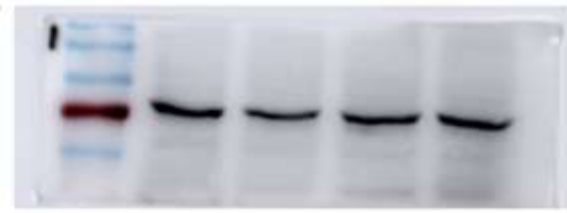

P-AKT

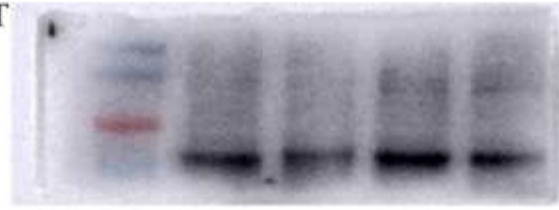

AKT

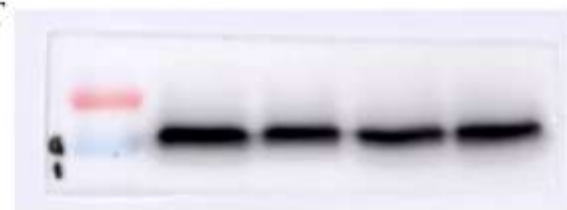

$\beta$ -actin

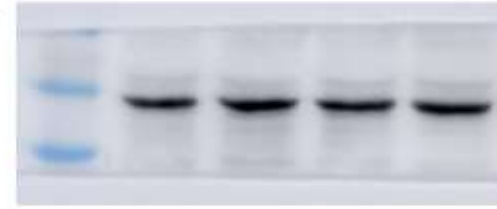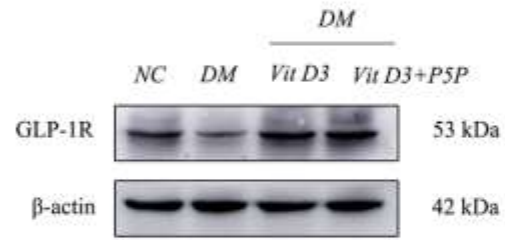

GLP-1R

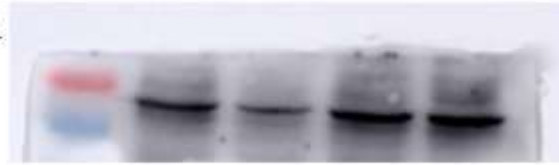

$\beta$ -actin

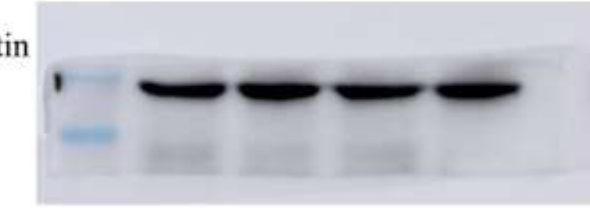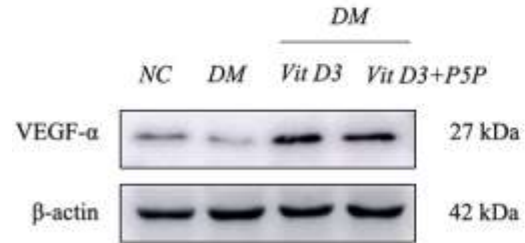

VEGF- $\alpha$

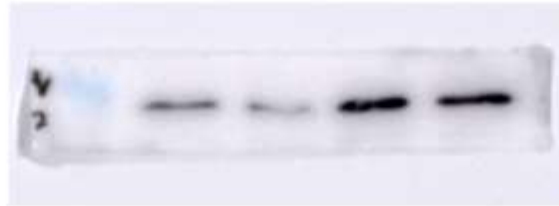

$\beta$ -actin

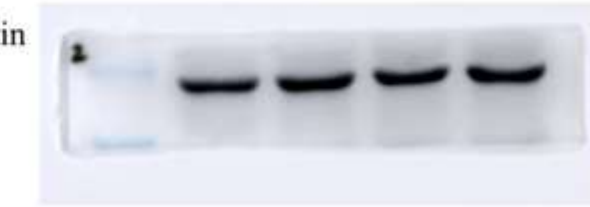

Supplement: Supporting Information 6 — Figure S4: images of WBs in group one. [file 8217035.f6.pdf]

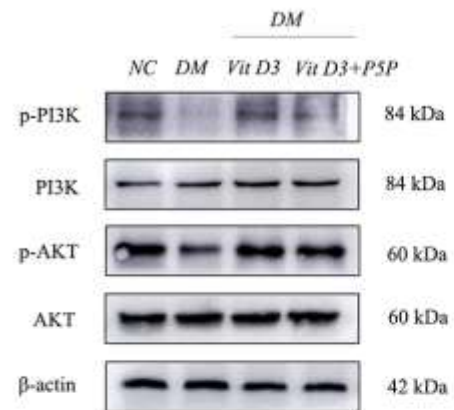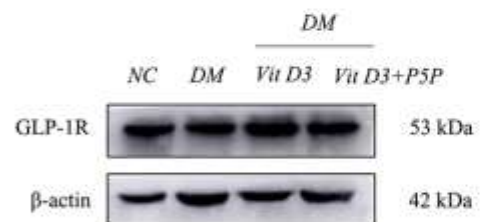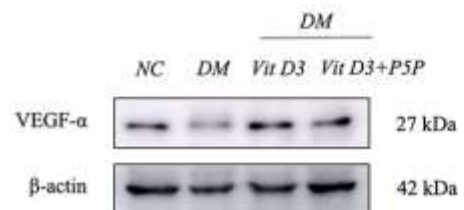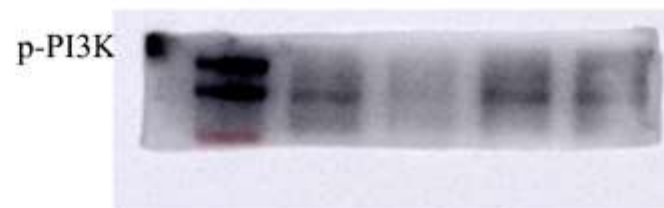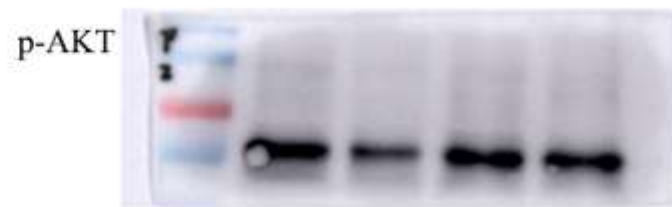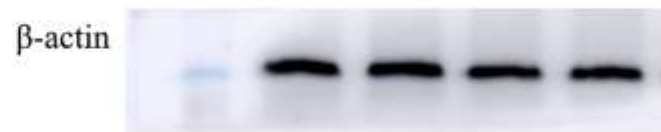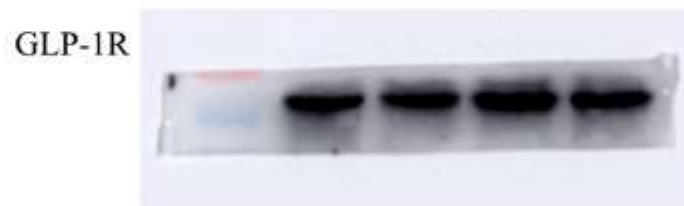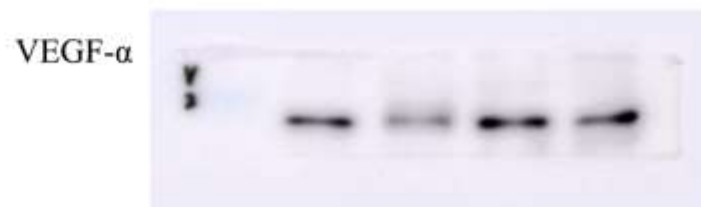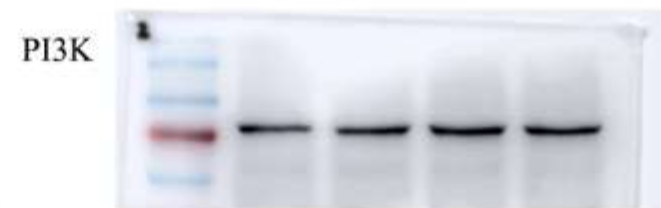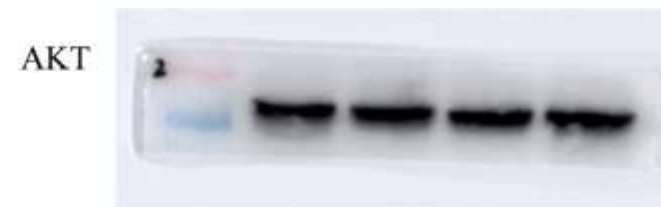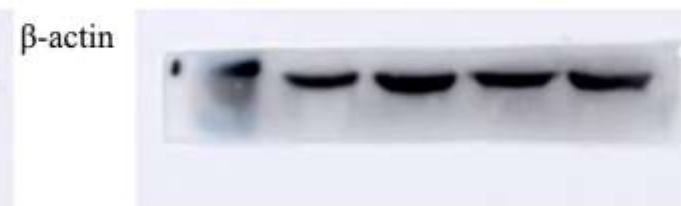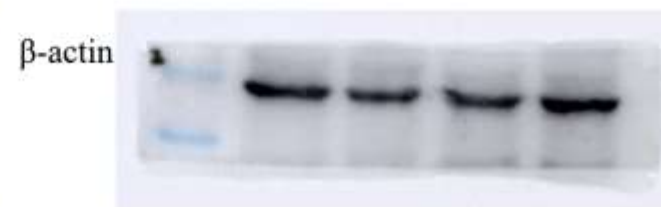

Supplement: Supporting Information 7 — Figure S5: images of WBs in group two. [file 8217035.f7.pdf]

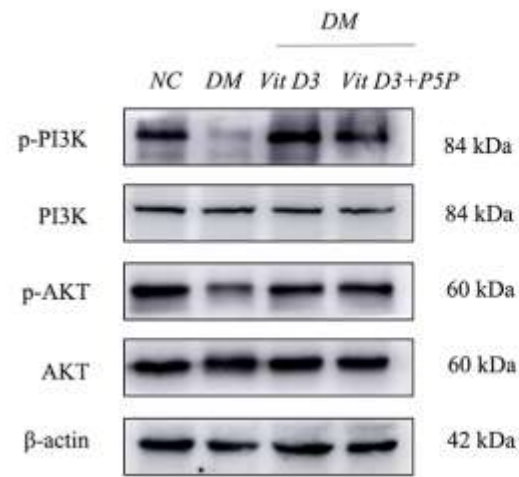

p-PI3K

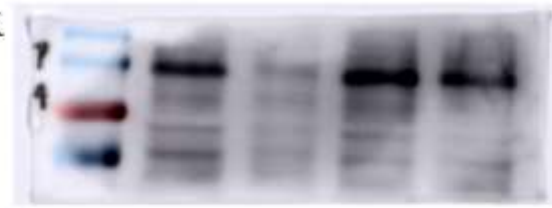

PI3K

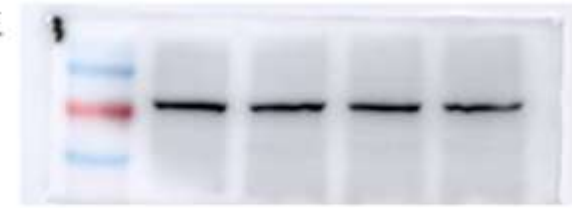

p-AKT

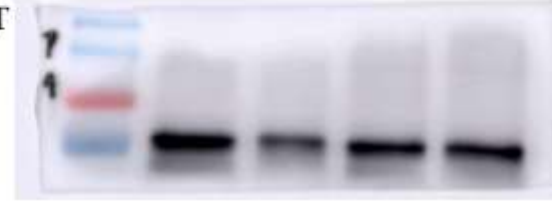

AKT

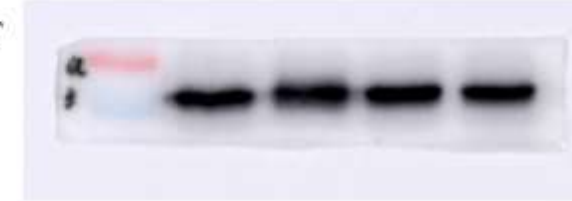

$\beta$ -actin

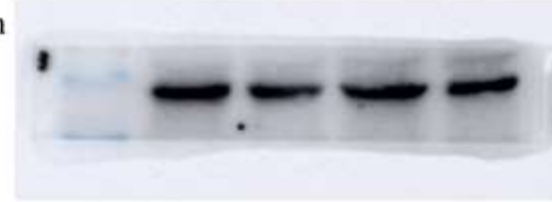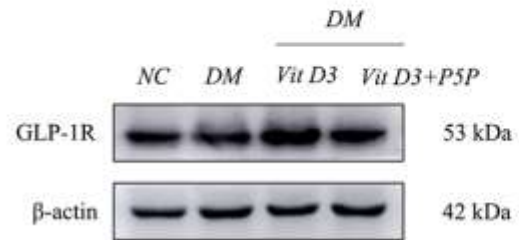

GLP-1R

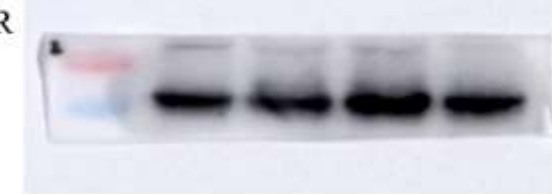

$\beta$ -actin

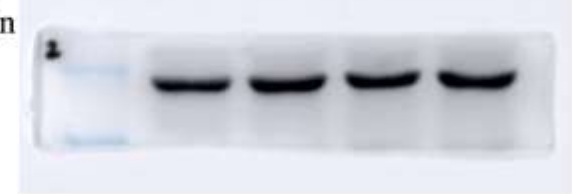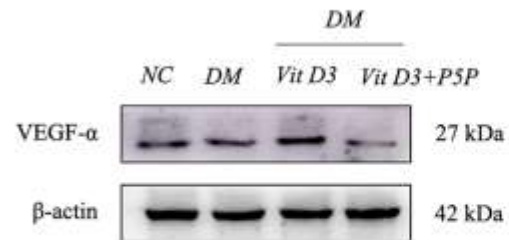

VEGF- $\alpha$

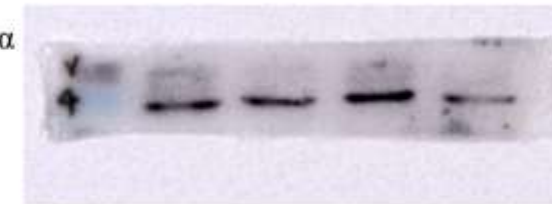

$\beta$ -actin

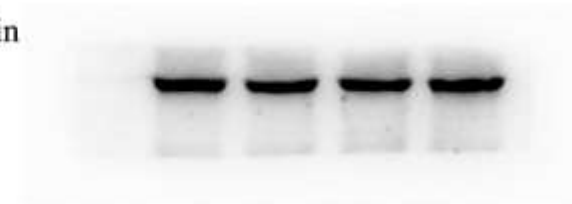

Supplement: Supporting Information 8 — Figure S6: images of WBs in group three. [file 8217035.f8.pdf]
