# Supplementary material for: 1,25-D3 Protects Diabetic Brain Injury Through GLP-1R/PI3K/Akt Pathway by Experimental and Molecular Docking Studies
Source: Mediators Inflamm. 2025 Mar 7;2025:8217035. doi: 10.1155/mi/8217035 (PMC11986256; doi:10.1155/mi/8217035)
Supplement: Supporting Information 3 — Table S1: the CT value of PCR and actual concentrations. [file 8217035.f3.pdf]

A	B	C	D	E
		13.804	10.008	7.599
△△ct		0.847	-0.725	-1.768
		0.013	0.427	0.371
		3.244	-0.303	-0.799
		-0.847	0.725	1.768
负△△ct		-0.013	-0.427	-0.371
		-3.244	0.303	0.799
2-△△ct		0.556	1.653	3.406
		0.991	0.744	0.773
		0.106	1.234	1.739
Group2	NC	DM	1,25-D3	1,25-D3+P5P
GLP1-R	1.000	0.556	0.991	0.106
ICAM-1	1.000	1.653	0.744	1.234
VCAM-1	1.000	3.4061082	0.7730959	1.7393406

Group2.GLP-1R ICAM-1 and VCAM-1 | Group3.GLP-

A	B	C	D	E
GLP1-R	1.000	0.173	0.703	0.494
ICAM-1	1.000	1.213	0.476	1.164
VCAM-1	1.000	1.613215077	0.551	1.550
	NC3	DM3	1,25-D3	VitD+P5P 3
GLP1-R	1.000	0.228	0.875	0.509
	1.000	0.556	0.991	0.106
	1.000	0.173	0.703	0.494
ICAM-1	1.000	1.114	0.870	1.255
	1.000	1.653	0.744	1.234
	1.000	1.213	0.476	1.164
VCAM-1	1.000	3.585	0.737	1.857
	1.000	3.406108	0.773096	1.739341
	1.000	3.22643	0.551063	2.43194

Group2.GLP-1R ICAM-1 and VCAM-1 | Group3.GLP-

A	B	C	D	E
2-△△ct		0.782	1.718	
		4.979	4.002	
		1.078	2.062	
Group1	NC	DM	1,25-D3	1,25-D3+P5P
eNOS	1.000	0.781699899	4.979335077	1.078399314
Vegf-α	1.000	1.717906545	4.001870804	2.061748553

Group2.GLP-1R ICAM-1 and VCAM-1 / Group3.GLP-1R ICAM-1 and VCAM-1 / Group1.eNOS and Vegf-α

A	B	C	D	E	F	G
	18.732	25.130	21.663			
	17.958	26.127	22.434			
△ct		8.360	5.222			
		8.865	4.216			
		6.398	2.931			
		8.169	4.475			
△△ct		0.505	-1.005			
		-1.962	-2.290			
		-0.191	-0.746			
		-0.505	1.005			
负△△ct		1.962	2.290			
		0.191	0.746			
2-△△ct		0.704	2.007			
		3.895	4.892			
		1.142	1.677			
Group2	NC	DM	1,25-D3	1,25-D3+P5P		
eNOS	1.000	0.704476266	3.894806245	1.141511176		
Vegf-α	1.000	2.007270655	4.892251622	1.677405063		
