# Supplementary material for: 1,25-D3 Protects Diabetic Brain Injury Through GLP-1R/PI3K/Akt Pathway by Experimental and Molecular Docking Studies
Source: Mediators Inflamm. 2025 Mar 7;2025:8217035. doi: 10.1155/mi/8217035 (PMC11986256; doi:10.1155/mi/8217035)
Supplement: Supporting Information 4 — Table S2: the relative concentrations of mRNA. [file 8217035.f4.docx]

Table S1 The actual concentrations of genes/mRNA of these molecules (GLP1-R/ ICAM-1/ VCAM-1/ e-NOS/ VEGF-α)

|  | NC3 | DM3 | 1,25-D3 | 1,25-D3+P5P |
| --- | --- | --- | --- | --- |
| GLP1-R | 1.000 | 0.228 | 0.875 | 0.509 |
|  | 1.000 | 0.556 | 0.991 | 0.106 |
|  | 1.000 | 0.173 | 0.703 | 0.494 |
| ICAM-1 | 1.000 | 1.114 | 0.870 | 1.255 |
|  | 1.000 | 1.653 | 0.744 | 1.234 |
|  | 1.000 | 1.213 | 0.476 | 1.164 |
| VCAM-1 | 1.000 | 3.585 | 0.737 | 1.857 |
|  | 1.000 | 3.406 | 0.773 | 1.739 |
|  | 1.000 | 3.226 | 0.551 | 2.432 |
| e-NOS | 1.000 | 0.704 | 3.895 | 1.142 |
|  | 1.000 | 0.782 | 4.979 | 1.078 |
|  | 1.000 | 0.861 | 4.637 | 0.880 |
| VEGF-α | 1.000 | 2.007 | 4.892 | 1.677 |
|  | 1.000 | 1.718 | 4.002 | 2.062 |
|  | 1.000 | 2.059 | 4.528 | 1.384 |
